# Combining targeted and immunotherapy: BRAF inhibitor dabrafenib (D) ± the MEK inhibitor trametinib (T) in combination with ipilimumab (Ipi) for V600E/K mutation-positive unresectable or metastatic melanoma (MM) 

**DOI:** 10.1186/1479-5876-13-S1-K8

**Published:** 2015-01-15

**Authors:** Igor Puzanov

**Affiliations:** 1 Vanderbilt-Ingram Cancer Center, Vanderbilt University, Nashville, Tennessee, USA

## Introduction

Dabrafenib, trametinib, and Ipilimumab are each indicated for treatment of patients (pts) with metastatic melanoma (Dabrafenib+trametinib in *BRAF* V600 mutation–positive metastatic melanoma). Dabrafenib and trametinib can be safely combined and prolong progression-free survival compared with monotherapy. Combining Dabrafenib+trametinib with the CTLA-4 antibody Ipi has the potential to improve treatment outcomes, but the safety profile is unknown. A recent report suggested caution in combining the BRAF inhibitor vemurafenib (V) with Ipilimumab; V+Ipilimumab resulted in G3 elevations of ALT in 6/10 pts leading to study discontinuation (NEJM2013 368; 14). The present study was designed to characterize the safety of Dabrafenib±trametinib+Ipilimumab, select recommended phase 2 doses (RP2Ds), and report efficacy.

## Methods

Pts with stage IIIc/IV *BRAF* V600E /K mutation–positive MM and ≤1 prior treatments are eligible. Dose escalation occurs in cohorts of 3-6 pts followed by expansion (≤30 pts) at the RP2D. At data cutoff (Nov 8, 2013), 10 pts were enrolled: 4 received D+Ipi (doublet), 2 received D only (withdrawn before Ipi treatment), and 4 received D+T+Ipi (triplet).

## Results

Median age of the 10 pts was 59.5 y (range, 32-75 y). **Doublet**: D 150 mg bid + Ipi 3 mg/kg q3w × 4 doses was well tolerated and selected as RP2D. No G3/4 ALT elevations or dose-limiting toxicities (DLTs) were observed. The most frequent adverse events (AEs; ≥2) were chills, fatigue, hand-foot syndrome, pyrexia, and maculopapular rash. Of 4 pts, 2 are ongoing and 2 stopped treatment (disease progression). Pts are currently being enrolled at this dose level in the expansion. **Triplet**: At current doses (D 100 mg bid/T 1 mg qd+Ipi 3 mg/kg q3w × 4), 2 out of 7 patients developed G3 colitis complicated by perforation.The triplet combination enrollment was therefore stopped. The most frequent AEs (≥2) were pyrexia, chills, arthralgia, insomnia, and maculopapular rash. One pt had G4 renal insufficiency that reversed rapidly.

## Conclusions

To date the combinations of D+Ipi and D+T+Ipi appear to be tolerable and have not been associated with significant hepatotoxicity in MM, suggesting differences between BRAF inhibitors when combined with Ipi. However, combination of dabrafenib+trametinib+Ipilimumab was stopped early after 2 out of 7 patients developed colon perforation soon after initiating Ipilimumab therapy.

